# Superiority of combining psychopathology and the second-generation cognitive science: a discussion of Kristopher Nielsen’s pluralist approach

**DOI:** 10.3389/fpsyg.2025.1578600

**Published:** 2025-07-01

**Authors:** Yaming Shang, Da Dong, Qingming Liu, Jiali Li

**Affiliations:** ^1^Department of Mathematics, Shaoxing University, Shaoxing, China; ^2^Center for Brain, Mind and Education, Shaoxing University, Shaoxing, China; ^3^Department of Psychology, Shaoxing University, Shaoxing, China

**Keywords:** cognitive sciences, 4E cognition, enactivism, psychopathology, mental disorders, explanatory pluralism

## Abstract

In recent years, advancements in the second-generation cognitive science have significantly contributed to interdisciplinary progress at the intersection of psychopathology and cognitive sciences. This article critically examines Kristopher Nielsen’s 3E framework—embodied, embedded, and enactive—which challenges traditional conceptual models of mental disorders. By emphasizing the dynamic interplay among the brain, body, and environment, the framework addresses the limitations of existing approaches, offering a more comprehensive and ecologically valid perspective on psychopathology. In addition, the article proposes directions for future research, underscoring the significance of pluralism in the explanation of mental disorders and exploring the possibility of integrating extended cognition into the existing framework. These theoretical developments enhance multifaceted understandings of mental disorders, refine classification and explanatory methodologies, and inform the development of evidence-based, targeted treatment strategies. Collectively, these insights aim to advance the field of psychopathology toward a more integrated, inclusive, and practice-oriented realm, providing a robust theoretical foundation for innovative clinical approaches to mental health.

## 1 Introduction

Psychopathology examines mental illness and abnormal behavior, increasingly intersecting with cognitive science. Cognitive science, as an interdisciplinary field, has undergone two major theoretical evolutions. Initially, it focused on symbolic representation and computational processing, conceptualizing cognition as an internal, mechanistic operation. Conversely, the second generation emphasizes the dynamic interrelation of brain, body, and environment, characterizing cognition as embodied, embedded, enactive, and extended (4E cognition) (Chen et al., 2023; Clark and Chalmers, 1998; Lakoff and Johnson, 1999; Shapiro and Spaulding, 2014; Varela et al., 2017; Walter, 2010). This paradigm shift provides a more comprehensive framework, capturing the multidimensional nature of the mind.^1^

When engaging with the concept of mental disorder in everyday discourse, individuals often exhibit confidence in defining the term and providing examples. Yet, there exists no universally accepted classification system or unified theoretical framework, particularly in the context of cognitive science. In *Embodied, Embedded, and Enactive Psychopathology*: *Reimagining Mental Disorder*, Kristopher Nielsen critiques conventional approaches to psychopathology and advocates for the adoption of a 3E framework—embodied, embedded, and enactive cognition. Nielsen argues that the second-generation cognitive science provides essential tools for rethinking mental disorders by situating them within a dynamic and relational model of cognition. This perspective deepens explanatory power while enhancing ecological validity by bridging insights from laboratory studies with the complexities of real-world clinical practice. By foregrounding the lived experience of patients and the intricate causal pathways underlying mental illness, Nielsen’s 3E framework lays a compelling foundation for the development of innovative and context-sensitive intervention strategies (Nielsen, 2023).

Nielsen advocates for a 3E cognitive framework rather than a 4E framework due to fundamental concerns about extracranial cognition associated with extended cognition. He argues that extended cognition conflicts with the core principles of embodied, embedded, and enactive cognition. By locating aspects of the mind in the external environment, extended cognition risks eroding the autonomy and self-contained nature of the cognitive subject. Moreover, it may lead to an unbounded expansion of normativity, allowing for an indeterminate array of values and norms without clear criteria for prioritization. Practically, this framework complicates the analysis of individual psychopathology by introducing ambiguities regarding subject-system boundaries, rendering interpretations both unclear and operationally impracticable.

Adopting a comprehensive perspective, we identify a core task of psychopathology and examine existing conceptual models of mental disorders. We then introduce the theoretical underpinnings and integrative framework of 3E psychopathology. Finally, we trace the evolution of psychopathology within cognitive science, elaborate on our position, and outline aspirations for advancing psychopathology and improving mental health treatment.

## 2 The core tasks of psychopathology research

Within the field of psychopathology, precisely articulating a conceptual definition of mental disorders is an urgent and core task (Nielsen, 2023). To further explore and develop the concept of mental disorders. There are three key questions:

- Are mental disorders something you get or something you do?
- Does a mental disorder exist inside someone’s brain, or is it dispersed across their brain, body, and environment?
- The final question to an example of the need for conceptual work in psychopathology: are mental disorders defined by brute facts or by social norms and values? (pp. 6–7)

Through discussion, we concluded that these three issues can be encapsulated in three succinct statements: mental disorders are inherently complex, as they manifest both individual behaviors and involuntary elements; although based on factual evidence, mental disorders are influenced by normative factors at a functional level; and from a systematic perspective, mental disorders represent a complex network of interacting causal factors within the brain–body-environment system.

Definitions of mental disorders, whether explicit or implicit, directly influence our understanding and practice of classification, explanation, and treatment. These three tasks form the core of psychopathological research. The classification task seeks to impose order on the diversity of behaviors and experiences (Berenbaum, 2013); the explanation task involves formulating and validating theoretical hypotheses to enhance our understanding of mental disorders (Haig, 2014); and the treatment task focuses on developing and validating effective interventions, whether pharmacological, psychotherapeutic, or otherwise. A well-considered classification system and a valid explanation framework provide a solid foundation for treatment efforts. Current research indicates that the traditional three-task model is incomplete, and the introduction of a four-task model through the inclusion of conceptual tasks demonstrates greater strengths. The conceptualization of mental disorders underpins our work in classification, explanation, and treatment. In addition, all of these tasks make a pluralistic commitment that can provide diverse perspectives and insights for understanding and addressing the complex entity of mental disorders.

### 2.1 Nielsen’s current conceptual model

Nielsen’s existing conceptual model can be categorized into two primary frameworks: structure-oriented concepts and norm-oriented concepts. These frameworks enhance our understanding of the nature of mental disorders; however, they do not negate the necessity for conceptual refinement and the development of improved models.

Structure-oriented concepts primarily focus on the nature of mental disorders and their existence within a physical or causal framework, akin to analyzing the nature and composition of an object. Haslam (2002) proposes a conceptual taxonomy that effectively integrates various perspectives on the structural nature of psychopathology, ultimately advocating for conceptual pluralism—the notion that different mental disorders may possess distinct structural characteristics. While this taxonomy is valuable for integrating diverse viewpoints, it also encounters certain challenges. Specifically, the lack of definitive cut-off points in the classification process can lead to the oversimplification of mental disorders, thereby neglecting the complexity of individual differences and environmental factors. This oversimplification not only complicates further research but also poses challenges for the implementation of effective treatment.

In contrast, norm-oriented concepts examine why certain phenomena should be regarded as obstacles from an evaluative and functional perspective. These concepts aim to establish a framework for “conceptual validity,” which refers to the ability to accurately distinguish between normal and dysfunctional functioning (Wakefield, 2014). This process can be likened to determining whether a specific behavior is abnormal. However, the evaluation of such concepts often lacks clear, objective criteria, and the evaluation process is inevitably influenced by the invasion of subjectivity. This subjectivity, which can vary according to cultural and social contexts, presents challenges for diagnosis and treatment, hindering in-depth exploration of the mechanisms and causes of mental disorders (Jefferson, 2014).

### 2.2 The 3E psychopathology approach

Given the urgent need for a conceptualization of mental disorders and the limitations of existing conceptual models, the 3E psychopathology framework has emerged as a novel approach to understanding these disorders through an embodied, embedded, and enactive view of human functioning. The fundamental concepts of 3E cognition were introduced at the beginning of this article. This section focuses on a synopsis of previous research on the 3E framework and discusses how it can be further refined based on this research to significantly contribute to the treatment of mental.

Fuchs (2017) presents an embodied perspective in his research, introducing the significant concepts of dual orientation and circular causality. Dual orientation challenges the traditional binary distinction between psychology and physics, positing that conscious experiences and the physical states of the brain and body are manifestations of the same phenomenon observed from different perspectives. In understanding mental disorders, the experience of consciousness integrates with the physical state of the brain and body, and the interaction between these elements constitutes a comprehensive understanding of mental disorders. Circular causality encompasses both horizontal and vertical dimensions (Fuchs, 2023). Horizontal circular causality reflects the dynamic interactions between an individual and his/her environment; while vertical circular causality pertains to the interactions among various hierarchies within an individual, from molecules to cells to organs to the entire body. This dimension emphasizes how smaller-scale structures are constrained and influenced by larger-scale organizations, such as gene expression, which may be regulated by an individual’s overall physiological and psychological state. Fuchs argues that mental disorders disrupt normal vertical and horizontal circular causality, resulting in atypical perceptual and reaction patterns.

De Haan (2020) posits that mental disorders represent systematic deviations in the process of meaning construction across four dimensions: physiological, experiential, socio-cultural, and existential. For instance, individuals with anxiety disorders may exhibit an exaggerated assessment of environmental threats (the experiential dimension), which could be closely linked to the physiological stress response (the physiological dimension), sociocultural pressures (the socio-cultural dimension), and confusion regarding the meaning of their existence (the existential dimension). While this framework offers a multidimensional perspective and effectively describes the characteristics and manifestations of mental disorders, it requires enhancement in explaining how these disorders develop and persist, as well as in tailoring approaches to individual experiences. Maiese (2021) similarly characterizes mental disorders as disruptions in meaning construction and introduces the concept of habits to elucidate the formation and maintenance of these disorders. But, one limit of Maiese’s approach might be the potential overemphasis on social expectations. Social expectations are dynamic; a particular behavior may be deemed appropriate in some contexts while considered socially inappropriate in others. This variability can result in misinterpretations of certain normative behaviors.

Nielsen (2023, p. 97) concludes all three views seem to agree that: (1) mental disorders can in some sense be understood as disruptions to sense-making; (2) this allows for a holistic and integrated/embodied view of mental disorder; (3) this also allows for a middle way between naturalist and normative views.

By utilizing the core conceptual tools of 3E cognition, a framework for 3E psychopathology can be established. Its specific components are presented in Table 1.

**Table 1:** The core conceptual tools of the 3E cognition framework.

Conceptual tools	Main contents
Organizational causality, constitution, and dual aspectivity	It is essential to consider the organization and composition of individuals within their broader environment. It should also overcome the dualism of psychology and physics by analyzing the phenomenon of mental disorders from multiple levels through a dual orientation.
Naturalized normativity	Drawing from the processes of self-maintenance and adaptation in life, it is posited that norms and values emerge naturally and are closely linked to individual survival and development needs (Thompson, 2007).
Cultural embeddedness	It emphasizes the formation and development of individual meaning construction and behavioral patterns within specific cultural contexts. The values, beliefs, and social norms of different cultures significantly influence the perception and expression of mental disorders, as evidenced by varying cultural attitudes toward emotional expression. Incorporating this factor into research can mitigate cultural bias and facilitate the development of culturally adaptive treatment programs.
Thoroughgoing affectivity	Emotion is intricately linked to cognition and serves as a crucial force in meaning construction and behavior motivation (Krueger and Colombetti, 2018). In individuals with depression, negative emotions can significantly distort perceptions and interpretations of the world, thereby exacerbating symptoms. Understanding the role of emotion in this context could inform the development of treatments, such as training in emotional regulation skills.
A developmental perspective	Emphasis is placed on the influence of an individual’s history and developmental processes—including evolutionary, sociocultural, and individual life cycles—on current psychological states. Consequently, targeted prevention and intervention strategies can be implemented.
Demand for pluralism	A pluralistic approach that encompasses diverse conceptual definitions, classification methods, interpretive models, and therapeutic strategies is recommended for both research and treatment. Various mental disorders necessitate distinct strategies, promoting the flexible selection and integration of approaches to enhance effectiveness.

Leveraging these tools, 3E cognition emerges as a valuable perspective for explaining mental disorders. The original 3E conceptual model was developed from earlier structurally oriented and normatively oriented classification models. Structurally, the functional model in 3E psychopathology serves as a flexible concept that summarizes the intricate evolutionary history, cultural adaptation, and learning into a basic framework illustrating individuals’ efforts to survive and thrive. Mental disorders are viewed as dysfunctional patterns of constructing meaning that individuals tend to adopt. These patterns are recognized as intricate, multi-layered structures with interrelated causal networks present in brain–body-environment systems (Nielsen and Ward, 2018). Different from traditional disease models, mental disorders under the 3E framework are not simply caused by a single cause, but the result of the interaction of multiple factors. For example, in anxiety disorders, multiple factors may be involved, such as genetic factors, an individual’s early life experiences, current life stress, and abnormal regulation of the brain’s neurotransmitter system, which create a vicious cycle in the individual’s meaning-making process, leading to the persistence of anxiety symptoms. This structural understanding emphasizes the complexity and systematicness of mental disorders, which require comprehensive consideration of factors at multiple levels. From the normative point of view, mental disorders are the result of the serious contradiction between the individual’s meaning construction model and its functional norms, which will interfere with the normal function and quality of life of the individual (Thornton, 2000). In determining whether a behavior or mental state is a mental disorder, it is necessary to consider the individual’s unique background and functional needs. Normative judgments emphasize the importance of individual differences and situational factors and avoid simply diagnosing mental disorders based on statistical criteria or societal perceptions. These two aspects are also known as the skeleton of the 3E conceptual model.

## 3 Discussions

In the rapidly evolving field of psychopathology, several key elements significantly influence our understanding, exploration, and treatment of mental disorders. The development of psychopathology has been lengthy and complex, with its gradual integration into cognitive sciences emerging as an inevitable trend. This discussion will be structured around three primary aspects: First, we will examine the concept of “development” in the evolution of psychopathology and analyze how it shapes the current research landscape. Second, we will expand the conceptualizations, classifications, explanations, and treatment approaches from a pluralistic perspective, thereby invigorating psychopathology through their interactions. Third, we will focus on the progressive transformation from the 3E model to the 4E model, exploring the profound implications of this expansion for deepening the understanding of psychopathology and extending the boundaries of practice. Through a detailed analysis of these three points, we aim to illuminate the driving forces and challenges behind the vigorous development of psychopathology, providing a more robust theoretical foundation for future research and clinical applications.

### 3.1 The problem of “development” in psychopathology

Since the inception of psychopathology, research has shifted from mere symptom descriptions to the exploration of underlying mechanisms. Early studies primarily focused on external behaviors and symptoms, with diagnoses relying on subjective judgment and clinical experience. However, with advancements in science and technology and the diversification of research methods, there has been a deeper exploration of the biological, psychological, and sociological factors contributing to mental disorders (Berenbaum, 2013).

Nevertheless, significant challenges remain in the field’s current development. First, the heterogeneity of mental disorders is exceptionally high; symptomatic manifestations, pathogenesis, and treatment responses can vary significantly among individuals with the same disorder, complicating the formulation of uniform diagnostic criteria and effective treatment programs (Kendler, 2012). Additionally, there are gaps in our understanding of complex disorders such as schizophrenia and bipolar disorder, whose etiology and pathogenesis are not yet fully elucidated.

In our view, the advancement of psychopathology should emphasize interdisciplinary integration. Beyond traditional fields, there should be active collaboration with emerging disciplines such as computer science and artificial intelligence (Insel and Cuthbert, 2015). Furthermore, the implementation of large-scale longitudinal studies should be applied to monitor the long-term progression of mental disorders, allowing for a deeper understanding of their dynamic changes and the formulation of more targeted interventions. Additionally, we emphasize the importance of establishing a standardized research framework. This includes creating unified data collection standards for mental disorders that encompass symptom assessment, genetic testing, and brain function imaging to facilitate data integration and comparison across studies. Standardizing research design and statistical analysis methods will enhance the reliability and reproducibility of research results, thereby elucidating the etiology and pathogenesis of mental disorders and laying the groundwork for accurate diagnosis and treatment. Finally, future development must prioritize the experiences and participation of patients. Engaging patients and their families in research design and decision-making is essential to ensure that research questions align with their needs. In clinical practice, establishing a patient-centered diagnosis and treatment model that respects patients’ preferences and values, while enabling their active participation in treatment planning, is crucial (Johnstone, 2018). Moreover, feedback and suggestions can be gathered through patient organizations and community activities to continuously refine the theories and practices of psychopathology.

### 3.2 The plurality of conceptualization, classification, explanation, and treatment

According to Nielsen, pluralism has become a central pillar in the understanding and treatment of mental disorders, permeating every level of conceptualization, classification, explanation, and treatment. This pluralism reshapes our cognitive framework and practical approach to this complex field.

Conceptual pluralism reveals the richness of mental disorders by moving beyond a singular perspective. From a biological standpoint, certain mental disorders are viewed as manifestations of neurotransmitter imbalances or abnormal neural circuitry. The psychological dimension emphasizes factors such as cognitive biases, early experiences, and psychological defense mechanisms. The socio-cultural aspect underscores the significant influence of the social environment, cultural values, and family upbringing on individual mental states, such as the distinct interpretations and coping mechanisms related to certain spiritual phenomena in various cultures. This conceptual diversity encourages the integration of multidisciplinary insights, allowing for a holistic understanding of mental disorders while avoiding one-sided attributions (Haslam, 2002).

In addressing classification issues, the 3E psychopathology framework advocates for classification pluralism and humility. Although traditional classification systems for mental disorders, such as DSM, have standardized the diagnostic process to some extent, they also encounter numerous challenges (Lilienfeld and Treadway, 2016; Zachar and Kendler, 2017). Classification pluralism posits that, due to the complexity of mental disorders as conceptualized by the 3E framework, multiple effective classification methods should be explored (Markon, 2013). In clinical practice, we often face ambiguous boundaries and individual differences in patients’ symptoms, complicating the application of a singular classification standard. Advocacy for diversified classification supports the development of multiple models based on varying theoretical foundations, clinical characteristics, and research objectives. For example, classification could extend beyond symptomatology to include etiology, disease progression, or levels of functional impairment. This approach facilitates a more nuanced understanding of the distinct types of mental disorders, enhances the accuracy and specificity of diagnoses, and lays a solid foundation for tailored treatment plans that better meet patients’ diverse needs.

An explanation consists of explicit or implicit assumptions, typically presented as a series of premises, a model, a theory, a narrative, or a classification of causal information, aiming to elucidate the origin or perpetual presence of a single phenomenon or a group of phenomena (Haig, 2014; Thagard, 2019). There can be multiple different ways of interpreting the various changes in human behavior and experience. Explanation is also a practical task (Potochnik, 2016). Different backgrounds bring different investigative tools, different interpretive purposes, and different audiences, which may lead to differences in the quality of explanations.

Explanatory pluralism plays a crucial role in connecting various approaches within psychopathology. The explanation of mental disorders can be examined from both research and clinical perspectives. From a research standpoint, a single-explanation model often fails to provide a comprehensive understanding. It is essential to explore diverse and integrated viewpoints across different concepts, analyze the causes and mechanisms of mental disorders from multiple dimensions, and investigate the complex causal relationships that form a holistic explanation (Clack and Ward, 2020). This process can be likened to assembling a jigsaw puzzle, where a complete picture emerges from various pieces.

The interpretative work conducted from a clinical perspective signifies the initial steps in therapeutic development and establishes a foundation for precision therapy. Patient responses to treatment can vary significantly due to their unique characteristics, symptomatology, and life backgrounds. Treatment pluralism encompasses various modalities, including medication, psychotherapy, physical therapy, and social support, emphasizing the need for flexible combinations tailored to the specific circumstances of each patient. For serious mental disorders, medication may be critical for symptom relief, while issues primarily influenced by psychological factors may benefit from therapies such as cognitive behavioral therapy, psychoanalysis, or humanistic therapy, which help patients reshape their cognition and address psychological trauma (Bruch, 2015; Macneil et al., 2012). Additionally, emerging technologies, such as transcranial magnetic stimulation, offer new hope for some patients. Furthermore, the involvement of social support systems, including family support and community rehabilitation, is vital for the long-term recovery and social integration of patients. The diversity of treatment options ensures that each patient receives the most suitable treatment plan, maximizing therapeutic effectiveness and promoting mental health recovery and social functioning.

### 3.3 From 3E to 4E: a possible expansion of Nielsen’s project

In contemporary psychopathology, integrating cognitive sciences has become a dynamic and promising trend, driving research and practical advancements. This collaboration improves understanding of cognitive structures, sensory processing, and behavioral patterns in mental disorders. The 3E framework—embodied, embedded, and enactive cognition—addresses the limitations of traditional approaches by emphasizing the role of physical state, environment, and interactions.

Enactivism underscores the constitutive role of interaction between individuals and their environments in the formation of meaning, thereby foregrounding agency and the patient’s active engagement in treatment. This framework fosters self-efficacy and supports recovery, marking a departure from passive models of care. In clinical practice, adopting the 3E perspective enables clinicians to assess patients comprehensively and devise individualized treatment strategies that attend to bodily health, modify environmental conditions, and facilitate positive processes of meaning-making conducive to recovery (Di Paolo, 2005; Nielsen and Ward, 2018). The enactivist framework provides a distinctive perspective on anxiety disorders by stressing the interconnectedness of symptoms with both personal and environmental contexts. Unlike traditional models that treat anxiety as the result of internal dysfunction, enactivism emphasizes that symptoms arise from ongoing, dynamic interactions between individuals and their environments, further shaped by factors such as personality, coping styles, and social feedback. This approach foregrounds both the contextual embeddedness and the self-referential nature of anxiety, acknowledging that anxiety not only reflects situational factors but also reveals personal vulnerabilities and concerns. Clinically, the enactive paradigm supports recognizing and interpreting nonverbal cues—like facial expressions, body posture, and muscle tension—as manifestations of anxiety, encouraging patients to notice and articulate their bodily sensations for greater self-understanding and improved anxiety management. As Glas (2020) argues, by situating anxiety within this broader interactive framework, enactivism aligns with clinical intuition and fosters a move away from reductionist biomedical models. This promotes a richer, more nuanced, and patient-centered understanding and treatment of anxiety disorders.^2^

While the 3E approach to cognition has marked significant progress, the extended dimension of 4E cognition stands to yield further advances. This extension holds that cognitive processes are not confined to the individual or their immediate environment but are fundamentally embedded in wider social and cultural contexts (Rowlands, 2010). The social dimension involves the integration of cognition within social networks—family, peers, and professional circles—where relations and shared practices modulate thought and action through mechanisms such as support, comparison, and prevailing norms. The cultural dimension emphasizes that cognition is shaped by traditions, values, and systems of belief, with education, media, and social learning playing critical roles in structuring mental processes. Finally, the technological dimension recognizes that cognition is mediated by an increasingly complex array of tools and technologies. These not only alter how information is gathered and processed but also reshape patterns of social interaction and the very development of cognitive capacities (Chen et al., 2024; Dong and Chen, 2025; Dong et al., 2025). This conception thus broadens the philosophical analysis of cognition, situating it within a social, cultural, and technological ecology.

The introduction of these extended dimensions allows the 4E cognitive framework to more comprehensively explain the complexities of cognition and behavior. Specifically, the 4E cognitive framework offers several potential advantages: First, it provides a more comprehensive explanation, as it accounts for not only the influence of the individual’s body and immediate environment but also social, cultural, and technological factors. This holistic approach enhances our understanding of individual cognitive and behavioral performance across various contexts (Steiner, 2023). Second, it facilitates more effective interventions by offering a theoretical foundation for developing personalized and comprehensive treatment programs. By incorporating multifaceted factors, clinicians can devise interventions that may be more effective, such as psychotherapy that integrates social support networks and cultural contexts. Finally, the 4E cognitive framework encourages interdisciplinary research, fostering collaboration among scholars in psychology, sociology, cultural studies, and technical sciences to explore the intricacies of cognition and behavior (Lassiter and Vukov, 2021). This multidisciplinary approach aids in understanding the causes and mechanisms of mental disorders.

## 4 Conclusion

The integration of psychopathology with cognitive sciences, alongside the development of the 3E psychopathology framework, constitutes a substantial advancement in the field, both theoretically and practically. These developments offer new avenues for achieving a comprehensive understanding of mental disorders and devising effective interventions, while also posing several challenges. It is crucial to adopt an open, innovative, and interdisciplinary approach to refine and advance the field of psychopathology, thereby enhancing its scientific rigor, precision, and humane aspects. Such an approach not only improves diagnostic accuracy and treatment outcomes for mental disorders but also enhances patients’ quality of life and reduces social prejudice and discrimination. We aspire to bring hope and rehabilitation to a greater number of individuals with mental disorders and to foster overall health and societal harmony.

## Data Availability

The original contributions presented in the study are included in the article/supplementary material, further inquiries can be directed to the corresponding authors.

## Footnotes

^1^The representational/information-processing approach and the 4E approach, these seemingly opposed theoretical frames need not mutually exclude each other, and may be complimentary. For example, Piccinini (2024) presents a 4EA cognitive framework that meaningfully integrates representation-based mechanisms with embodiment. He argues for a synthesis, not a dichotomy, between these perspectives. Similarly, de Vignemont (2018) offers an in-depth analysis of the body schema, showing how representational and embodied aspects of cognition can be reconciled within an integrated model. Her work specifically addresses cases such as anxiety, showing how perceptual miscalibrations in anxious individuals reflect both representational and embodied dynamics. These attempts at integration provide a more nuanced view of cognitive theory and underscore the value of considering both representationalism and 4E approaches as potentially complementary rather than strictly oppositional. Over the past decade, scientists and philosophers, such as Friston (2010) and Clark (2013), have promoted the predictive processing framework, based on free energy principle and active inference, to unify the two generations of cognitive science. We thank the reviewer for highlighting this significant theoretical nuance.

^2^Thanks to the reviewer for drawing our attention to this point and the relevant example.
